# Social media addiction, escapism and coping strategies are associated with the problematic internet use of adolescents in Türkiye: a multi-center study

**DOI:** 10.3389/fpsyt.2024.1355759

**Published:** 2024-02-08

**Authors:** Esen Yildirim Demirdöğen, Mehmet Akif Akinci, Abdullah Bozkurt, Büşranur Bayraktutan, Bahadır Turan, Sevil Aydoğdu, İlknur Ucuz, Elif Abanoz, Gülsüm Yitik Tonkaz, Ali Çakir, Hurşit Ferahkaya

**Affiliations:** ^1^ Department of Child and Adolescent Psychiatry, Atatürk University Medicine Faculty, Erzurum, Türkiye; ^2^ Department of Child and Adolescent Psychiatry, Karadeniz Technical University Medicine Faculty, Trabzon, Türkiye; ^3^ Department of Child and Adolescent Psychiatry, İnönü University Medicine Faculty, Malatya, Türkiye; ^4^ Department of Child and Adolescent Psychiatry, Cumhuriyet University Medicine Faculty, Sivas, Türkiye; ^5^ Department of Child and Adolescent Psychiatry, Giresun University Medicine Faculty, Giresun, Türkiye; ^6^ Child and Adolescent Psychiatry Unit, Erzurum Region Training and Research Hospital, Erzurum, Türkiye; ^7^ Department of Child and Adolescent Psychiatry, Dr. Ali Kemal Belviranlı Maternity and Children Hospital, Konya, Türkiye

**Keywords:** problematic internet use, social media addiction, prefering virtual life, virtual pleasure, escapism, coping strategies

## Abstract

**Introduction:**

The literature highlighted that problematic internet use can have detrimental consequences on individuals’ well-being. Social media addiction, escapism and coping skills are closely related to problematic internet use. However, no study has currently examined the relationship between problematic internet use and social media use, escapism and coping skills.

**Method:**

This multicenter cross-sectional designed study evaluated the relationships between problematic internet use and social media addiction, escapism, and coping skills in 508 adolescents (319 female) aged 14-18 years. In order to collect data, sociodemographic data form, Internet Addiction Scale, Social Media Addiction Scale, Escapism Scale and Kidcope Adolescent Version have been used. First, adolescents who scored ≥50 on the Internet Addiction Test were classified as the problematic internet use group; adolescents who scored <50 were classified as the control group. Then, the relationships between problematic internet use and social media addiction, escapism and coping skills were evaluated.

**Results:**

The results showed that problematic internet use was associated with duration of social media use, impairment in social media-related functionality, preferring virtual life, and virtual pleasure, escapism, avoidant and negative coping strategies.

**Conclusion:**

These findings may provide an empirical basis for problematic internet use prevention and intervention in adolescence.

## Introduction

Problematic internet use is defined as individuals excessively and uncontrollably using the internet, resulting in adverse effects on their daily life functioning (1). This pattern of use signifies a shift from primary social and personal responsibilities to a state of neglect or even danger. Problematic internet use is commonly associated with negative outcomes, such as social isolation, occupational or academic underachievement, psychological distress, family problems, and physical health issues (2).

The prevalence of problematic internet use can vary across countries and age groups (3–5). Nevertheless, numerous research studies emphasize that this issue is becoming an increasing public health concern (6). A score of 50 or higher on the Internet Addiction Scale is typically considered an indicator of problematic internet use (7–9). The variations in problematic internet use prevalence across studies using the same instrument, like the Internet Addiction Scale, can be attributed to cultural differences, demographic factors, methodological variations, the evolving nature of technology, and individual psychosocial factors (10). Different populations may have varying degrees of exposure to and reliance on the internet. Age groups may have distinct patterns of internet use, with younger individuals typically more immersed in online activities. As a result, prevalence rates might vary across age groups. The criteria for problematic internet use may be interpreted differently in various cultural contexts. For example, what is considered excessive internet use or addiction in one culture might be perceived as normal or acceptable in another.

Problematic internet use can significantly impact an individual’s physical, mental, and social well-being, posing a threat to the general welfare of society (11, 12). Excessive internet use can lead to social isolation, strained interpersonal relationships, and decreased face-to-face interactions. This withdrawal from real-world social interactions may contribute to weakened social bonds, impacting community cohesion. Consequently, the societal impact of PIU stems from its potential to disrupt normal social dynamics, impair individual well-being, and contribute to broader issues such as decreased productivity and the deterioration of social relationships. Therefore, it is crucial for public health policies and treatment approaches to address this issue. Research and awareness-raising efforts can help individuals and families understand the risks associated with problematic internet use and contribute to the implementation of measures aimed at mitigating its effects (12).

Problematic internet use may relate to issues such as being addicted to social media, seeking escapism, and relying on certain coping mechanisms (13–16). Social media platforms provide an appealing avenue for individuals to satisfy their need for escapism, as users can adopt different identities or lifestyles in the virtual world. When this escape mechanism is employed to cope with emotional and psychological challenges, individuals may retreat from real-life problems, leading to problematic internet use. Furthermore, this behavior may undermine coping skills, as individuals may tend to use the internet as an escape route instead of addressing real-life issues (13). In this context, understanding the complex interplay between social media addiction, escapism, and problematic internet use is crucial for developing effective intervention strategies.

Despite numerous separate studies in the literature, there is a notable absence of research that concurrently investigates the relationship between problematic internet use, social media use, escapism, and coping skills within the same sample or a similar population. Given the potential variations in the prevalence of problematic internet use and its diverse clinical presentations/features (social isolation, academic or occupational impairment, psychological distress, sleep disturbances, neglect of basic needs, risk of cyberbullying, and financial consequences etc.), there is a pressing need for research examining these factors together in the same study. Such research could provide valuable insights into the underlying biopsychosocial mechanisms of problematic internet use and establish a foundation for improving the treatment and prevention approaches for this addiction. Filling this gap is an important step towards better understanding the effects of problematic internet use and enhancing the overall well-being of society.

## Method

### Setting and sample

This cross-sectional and multicenter (n=6) study was conducted between June and October 2023. Before starting the study, ethical approval was obtained from Atatürk University Faculty of Medicine Clinical Research Ethics Committee. Children aged 14-18 years and their families who were studying in high schools located in the study centers were informed by school staff. An informed consent form was filled out by the children and their parents who agreed to participate in the study. The following inclusion criteria applied: (a) the school board grants permission for the research; (b) participants are between the ages of 14 and 18; (c) written consent from adolescents and their parents stating that they agree to participate in the study. The data collection phase of the study was carried out in selected schools between June 2023 and October 2023. The administrators of the identified schools were informed about the study. At the designated times, the scales to be used in the study were distributed to adolescents accompanied by the responsible researcher of the province and a teacher. The children were asked to fill in the sociodemographic data form, Internet Addiction Scale, Social Media Addiction Scale, Escapism Scale and Kidcope Adolescent Version. Six provinces located in the northern, central, eastern regions of Turkey were included to represent various major geographical regions in Turkey to reduce sampling biases associated with single field surveys and to increase sample representativeness. The high schools were selected from schools in provinces using the simple random selection method. Adolescents in randomly selected schools were again selected using simple random sampling technique. The number of participants in each center was determined by weighting according to the population size of each province. In order to increase the reliability of this study, which was conducted on 3755446 children registered in the national education system of the provinces, the researcher aimed to reach at least 500 adolescents, which is well above the number required to represent the country, based on power analysis (The sample size was calculated to be 369 students as calculated using the Open EPI program.). The sample of the study consisted of 508 adolescent between the ages of 14-18 who were attending high school in Erzurum, Sivas, Malatya, Konya, Trabzon and Giresun provinces.

### Measures

Sociodemographic Data Form: Through this form, data such as adolescents’ gender, age, parental education level, family income level, social media usage status and daily social media usage time were collected.

Internet Addiction Test (IAT): The scale was developed by Young et al. in 1998. It is adapted from the “Pathological Gambling” criteria of DSM-IV and used to define the level of internet addiction. In the IAT, individuals are asked to rate how often they experience certain situations on a six-point Likert-type scale ranging from “never” to “always”. In this 20-item scale, higher scores indicate an increase in the severity of internet addiction. A score between 20-49 points indicates normal internet use and a score above 50 points indicates possible problematic internet use (17). The Turkish version of the scale was adapted by Bayraktar et al. and Cronbach’s alpha value was found to be 0.898 (18).

Social Media Addiction Scale (SMAS): The scale was developed by Orbatu et al., to measure social media addiction in adolescents in 2020. It is a 5-point likert and 13-item scale consisting of three sub-dimensions: impairment of functioning, preference for virtual life and enjoyment of virtual life. As the score increases, there is an assumption that the level of addiction is on the increase. The Cronbach’s alpha value of the scale was found to be 0.869 (19). Escapism Scale: The scale developed by Gao et al. in 2017, consists of four items measuring escape from the real world by using internet services or applications on personal devices (e.g. Social networking sites help me forget about the real world, Social networking sites help me get away from the problems and pressures in my life, etc.) (20). The Turkish version of the scale was adapted by Kırcaburun et al. and Cronbach’s alpha value was found to be 0.89. This form of the scale is a 7-point Likert-type scale and includes modified versions of the four items in the scale created by Gao et al. (e.g. Instagram helps me forget the real world, Instagram helps me get away from the problems and pressures in my life) (21). In our study, this scale was questioned not only for Instagram but also for social media use.

Kidcope Adolescent Version: It was developed to measure adolescents’ coping styles by Spirito et al. (22). It is an 11-item, 4-point Likert-type scale with three subdimensions: active, avoidant, and negative coping. Active coping involves cognitive restructuring, emotional regulation, social support, and problem-solving skills. Avoidant coping includes distracting, social isolating, suppressed thinking, and withdrawal strategies. Negative coping involves self-criticism and blaming others. The scale measures how often these coping strategies are used, with higher scores indicating more frequent use (22). The Turkish version was adapted by Bedel et al. The Cronbach alpha internal consistency values for the active coping, avoidant coping, and negative coping subscales of the scale were 0.72, 0.70, and 0.65, respectively (23).

### Data analysis

All statistical analyses were performed with SPSS for Windows Version 24.0. Adolescents who scored ≥50 on the IAT were classified as the “PIU” group; adolescents who scored <50 were classified as the “control” group. According to this classification, 253 (49.8%) of the 508 adolescents who participated in our study had problematic internet use, while 255 (50.2%) had normal internet use. Kolmogorov-Smirnov test was used to evaluate the conformity to normal distribution. Chi-square test was used to compare categorical data between PIU and control groups. Since the data were normally distributed, Student t test was used to compare numerical data. Pearson correlation analysis was used to assess relationships between variables, as the data were normally distributed. To identify possible risk factors for PIU, binary logistic regression analysis was used. The level of statistical significance was set at p < 0.05 for all of the analyses.

## Results

In our study, we first identified the groups with and without PIU according to IAT score. We compared these groups in terms of descriptive characteristics such as sociodemographic data, the daily social media usage time and the time since starting to use social media. Social media addiction, escapism and coping scale scores were then compared between the groups. In the PIU group, relationships between sociodemographic and descriptive characteristics and scale scores were analyzed. Finally, we performed logistic regression analysis to identify predictors of PIU.

There was no significant difference between PIU and control groups in terms of socio-demographic data such as gender, age, parental education level, family income, academic achievement, and social media use (p > 0.05). In the PIU group, the daily social media usage time and the time since starting to use social media were significantly higher (**Table 1**).

**Table 1:** The sociodemographic data of the sample.

	PIU Group n=253	Control Group n=255	t/Z/X^2^	p value
Age	15.67 ± 1.24	15.66 ± 1.19	0.037	0.971
Gender				
Gender Girl Boy	155 (61.3%) 98 (38.7%)	164 (64.3%) 91 (35.7%)	0.505	0.477
Mothers’ education (year)	10.41 ± 3.94	10.23 ± 4.15	0.479	0.632
Fathers’s education (year)	12.0 ± 3.49	11.53 ± 3.68	1.347	0.179
Family income			5.333	0.069
Low	15 (5.9%)	25 (7.7%)		
Middle	64 (25.3%)	76 (27.6%)		
High	174 (68.8%)	154 (64.7%)		
Academic achievement			3.065	0.216
Low	28 (11.1%)	23 (9.1%)		
Medium	147 (58.1%)	135 (47.9%)		
High	78 (30.8%)	96 (37.8%)		
Social media use			0.471	0.595
Yes	238 (94.1%)	236 (92.5%)		
No	15 (5.9%)	1987.5%)		
Daily social media usage time			19,044	**<0.001**
less than 1 hour	19 (7.5%)	37 (14.6%)		
1-3 hours	108 42.7%)	124 (48.8%)		
4-6 hours	93 (36.8%)	83 (32.7%)		
7 hours and more	33 (13%)	10 (3.9%)		
Time since social media use			9,001	**0.029**
less than 1 year	18 (7.1%)	21 (8.2%)		
1-3 years	104 (41.1)	124 (48.6%)		
4-6 years	93 (36.8)	92 (36.1%)		
7 years and more	38 (15%)	18 (7.1%)		

PIU, Pathological internet using Bold data, p<0.05 (signifcance).

In analyzing the Social Media Addiction Scale scores, all subscale scores (impaired functioning, preference for virtual life, virtual pleasure) were significantly higher in the PIU group. Escapism scale score was significantly higher in the PIU group. When coping scale scores were evaluated, avoidant coping and negative coping scores were significantly higher in the PIU group. Scores on the active coping subscale were not significantly different between groups (**Table 2**).

**Table 2:** Comparison of IAT, SMAS, Escapism and Kidcope scales between the PIU group and the control group.

	PIU Group n=253	Control Group n=255	t/Z/X^2^	p value
SMAS				
Impairment in functionality	15.26 ± 5.12	11.25 ± 4.01	9.831	**<0.001**
Preferring virtual life	14.98 ± 5.20	10.79 ± 4.35	9.856	**<0.001**
Virtual pleasure	5.74 ± 2.95	4.43 ± 2.51	5.382	**<0.001**
Escapism total scores	3.79 ± 1.9	2.57 ± 1.51	7.926	**<0.001**
Kidcope				
Active Coping	5.83 ± 2.65	5.97 ± 2.51	-0,584	0.559
Avoidant Coping	6.98 ± 2.59	6.16 ± 2.34	3.777	**<0.001**
Negative Coping	3.83 ± 2.05	2.77 ± 1.79	6.211	**<0.001**

PIU, Pathological internet using; IAT, Internet addiction test; SMAS, Social media addiction scale Bold data, p<0.05 (signifcance).

In the PIU group, there was a positive correlation between the IAT total score and the daily social media usage time, all subscale scores of social media addiction, escapism total score, and Kidcope avoidant and negative coping subscale scores (**Table 3**).

**Table 3:** Correlations between IAT, SMAS, Escapism and Kidcope scales in the PIU group.

Scales		Daily social media usage time	IAT total scores	SMAS Impairment in functionality	SMAS Preferring virtual life	SMAS Virtual pleasure	Escapism total scores	COPE Active Coping	COPE Avoidant Coping	COPE Negative Coping
Daily social media usage time	r	–								
	p									
IAT total scores	r	.215	–							
	p	**.001**								
SMAS Impairment in functionality	r	.083	.349	–						
	p	.186	**<.001**							
SMAS Preferring virtual life	r	.117	.451	.328	–					
	p	.062	**<.001**	**<.001**						
SMAS Virtual pleasure	r	.094	.146	.243	.305	–				
	p	.137	**.020**	**<.001**	**<.001**					
Escapism total scores	r	.043	.192	.249	.309	.295	–			
	p	.497	**.002**	**<.001**	**<.001**	**<.001**				
Kidcope Active Coping	r	-.047	-.084	-.001	-.009	.066	.003	–		
	p	.460	.181	.984	.886	.297	.961			
Kidcope Avoidant Coping	r	-.058	.179	.102	.328	.059	.199	.194	–	
	p	.353	**.004**	.106	**<.001**	.350	**.001**	**.002**		
Kidcope Negative Coping	r	-.013	.292	.249	.169	.183	.246	-.068	.124	–
	p	.835	**<.001**	**<.001**	.007	.003	**<.001**	.281	**.048**	

PIU, Pathological internet using; IAT, Internet addiction test; SMAS, Social media addiction scale; r, Pearson correlation coefficient Bold data, p<0.05 (signifcance).

According to the results of logistic regression analysis, it was observed that the duration of social media use over seven hours, high scores on the functional impairment and social life preference subscales of the social media addiction scale, high scores of escapism and negative coping strategies predicted the development of PIU (**Table 4**).

**Table 4:** Logistic regression analysis results for PIU.

Independent variables	OR	95% CI	p value
Age	.902	.754 -1.080	.262
Gender (reference: female)			
Male	1.645	1.036-2.612	.135
Daily social media usage time (reference less than hour)			
1-3 hours	1.474	.731 -2.971	.278
4-6 hours	1.438	.692-2.990	.330
7 hours and more	5.686	1.953-16.559	**.001**
Time since social media use (reference less than 1 year)			
1-3 years	.844	.381-1.869	.675
4-6 years	.899	.397-2.035	.798
7 years and more	1.260	.441-3.601	.666
SMAS Impairment in functionality	1.110	1.056 – 1.166	**>.001**
SMAS Preferring virtual life	1.134	1.072-1.199	**>.001**
SMAS Virtual pleasure	1.006	.925 -1.094	.884
Escapism total scores	1.201	1.049 -1.374	**.008**
Kidcope Avoidant Coping	1.010	.921-1.107	.836
Kidcope Negative Coping	1.178	1.049- 1.322	**.006**

PIU, Pathological internet using; SMAS, Social media addiction scale; OR, Odds Ratio; CI, Confidence interval Bold data, p<0.05 (signifcance).

## Discussion

In this study, the relationships between problematic internet use and social media addiction, escapism and coping skills in adolescents were evaluated. To the best of our knowledge, no previous study has done this. In the PIU group, impaired functioning associated with social media addiction, preferring virtual life, virtual pleasure, escapism, avoidant and negative coping were significantly higher. In the PIU group, the severity of PIU increased as all these variables increased. Social media use for more than seven hours a day, impaired social media-related functioning and preferring virtual life, escapism and negative coping predicted the risk of PIU. This research has implications for the conceptualization of PIU in adolescents.

### PIU and social media addiction

Studies have shown that there is a positive relationship between the duration of social media use and PIU (24, 25). Furthermore, social media use has been shown to be a predictor for IA (26, 27). In a study investigating the determinants of IA onset and persistence among adolescents, online activities, such as social media use, were found to be important determinants of IA onset and persistence (28). In a study evaluating IA risk factors among adult students, social media use was found to increase the risk of IA the most among all online activities (29). Social media use was found to increase the risk of IA by 3.2% in a study that evaluated risk factors for IA in adolescents (30). In our study, in line with the literature, we found that the duration of daily social media use was higher in the PIU group, the severity of problematic internet use increased as the time spent on social media increased, and the time spent more than seven hours predicted the development of PIU. We also found that impaired functioning, preference for virtual life and virtual pleasure associated with social media addiction were higher in the PIU group, the severity of problematic internet use increased as these variables increased, and these variables (except virtual pleasure) predicted the development of PIU. Although there are studies suggesting that social media addiction (SMA) is not a true addictive behavior but a secondary condition that emerges as a coping mechanism for negative life events (31), most studies show that SMA is a clinical condition and leads to impaired functioning (32–34). Despite these different views, there is a consensus that it is increasingly important to better understand the clinical features of SMA and PIU and that this should be based on criteria that do not risk over-pathologizing daily life habits (32, 34).

At this point, in addition to assessing the symptoms of social media addiction, assessing also functional impairment, which is a key criterion in many mental disorders, including gaming and gambling disorders (35) becomes important. To the best of our knowledge, no study has evaluated the relationship between problematic internet use and impairment in functionality associated with social media addiction. Our results that impairment in functionality associated with SMA increases the severity of PIU and predicts PIU suggest that it may be useful to evaluate the effect of risky behaviors related to social media on daily life and functionality in the prevention and management of PIU and to include them in the intervention plan. Social media facilitates social networking and interpersonal relationships and is preferred by people as a more comfortable social environment due to its nature that does not have many of the difficulties encountered in face-to-face communication. However, despite these advantages, studies have shown that a tendency to have more online friends and a preference for online communication are associated with an increased risk of IA (36, 37). This result is consistent with the result in our study that preferring virtual life is associated with PIU. Research has shown that communication pleasure is the strongest predictor of IA (38). It has been suggested that SMA, one of the most commonly used Internet applications for communication pleasure, is a risk factor for IA (29). In our study, the increase in the severity of PIU as the virtual pleasure associated with social media increases seems to be consistent with the literature. Our results suggest that in PIU assessment and intervention, there is a need for a better characterization of SMA and for specific counseling programs for SMA. Specifically considering the factors that predict PIU, such as social media use over 7 hours, impairment in functionality associated with SMA and preferring virtual life, we suggest that it would be important for intervention programs to cover the following points: Determine the time limit rather than whether or not to use social media, evaluate the impact of social media use on adolescents’ daily lives and adolescents’ motivation to use social media (preferring virtual life for communication, virtual pleasure, etc.) and developing specific solution skills for them.

### Escapism and PIU

Studies have shown a relationship between escapism and PIU (39, 40). A study examining escapism and internet addiction in senior high school students showed that psychological distress increased the tendency to escapism and escapism increased the tendency to internet addiction, causing negative effects on the individual’s daily life. The authors described this relationship as the Escapism-Addiction model (15). In a study conducted in university students, escapism was shown to mediate the relationship between emotional dysregulation and PIU (41). In our study, the escapism scale was used for social media use. In the PIU group, the level of escapism was higher. As the severity of escapism increased in the PIU group, the severity of problematic internet use also increased. In addition, higher escapism was predictive for PIU. Moreover, in the PIU group, escapism was positively associated with impaired social media-related functioning, preference for virtual life, and virtual pleasure. Thus, both SMA itself and escapism, the use of social media to alleviate emotional distress, increase PIU. Because of the role of escapism as an initiator, amplifier, and reinforcer of addictive behaviors, we believe it is very important to address escapism in PIU prevention and intervention.

Although escapism and avoidant coping are separate constructs, they are closely related. In the literature we noticed that escapism studies have been conducted specifically in the context of Internet gaming disorder, and the terms escape and avoidance are often used interchangeably. The researchers argued that if the meaning of these constructs is not communicated clearly enough, the interpretation of the findings will be difficult. Therefore, they suggested that the nature of escapism, especially as an avoidant coping strategy, should be clearly defined (42). In our study, while escapism refers to the use of social media to escape from reality, avoidant coping means that the individual uses coping strategies such as trying to forget the problem by doing other things, trying to solve the problem by staying alone, wishing the event had never happened, and trying to accept the situation by thinking that there is nothing else to do. Although they are closely related constructs, there is no research in the literature that examines the relationship between escapism and coping skills in PIU and the effect of these two constructs together on PIU. Therefore, we included coping skills including avoidant coping as well as escapism in our study.

### PIU and coping style

Many studies have investigated the relationship between coping strategies and internet addiction. Most of this research suggests that Internet addiction is associated with more negative coping and fewer positive coping strategies (43, 44). It was observed that the term negative coping strategies has different meanings in the literature and covers different strategies depending on the measurement tool used. In some studies, avoidance and negative coping strategies were evaluated separately; in others, both strategies were combined under the umbrella of negative coping and reflected a general statement. In a study examining Internet addiction, psychological distress, and coping responses, coping responses were categorized as maladaptive (rumination and acting out) and adaptive (resiliency, seeking social support, and self-care). This study has shown that high levels of rumination and low levels of self-care are the main factors that contribute to the development of IA in adolescents (13). In a study examining the moderating role of coping style in Internet addiction among adolescents, coping style was assessed on two dimensions: positive (e.g., “trying to find several different solutions”) and negative (“imagining that miracles will happen and the current situation can change”). In this study, negative coping was found to be positively associated with IA, and adolescents who used more negative coping strategies were more likely to develop IA (45). Negative coping styles, including self-blame, fantasy, and avoidance, were found to play an important role in IA in a study that examined negative life events, negative coping styles, and IA in middle school students (46). In line with the literature, we found that the PIU group used avoidant coping strategies (trying to forget by doing other things, trying to solve the problem by staying alone, wishing the event had never happened, trying to accept the situation by thinking that there was nothing else to do) and negative coping stretegies (blaming oneself by thinking that the problem was caused by oneself, blaming others for the problem, shouting and harming the environment by expressing anger) more frequently. Avoidant and negative coping skills were positively associated with PIU severity. In addition, negative coping skills predicted the development of PIU. In addition to other studies, we found in the PIU group that avoidant and negative coping were associated with escapism, and avoidant coping was associated with a preferring virtual life. Adolescents who used more negative coping strategies were more likely to have PIU. This may be explained by the fact that, as shown in previous studies (47, 48) adolescents with PIU prefer to use the internet to distract themselves from problems by using negative coping strategies in case of stress because the internet provides them with more controllable conditions, unlike the uncontrollable nature of the real world. In other words, adolescents may consider internet use as a coping mechanism to get away from stressors. That both avoidant and negative coping were associated with escape in PIU group supports that PIU is a negative coping strategy. Davis’s cognitive-behavioral theory of PIU suggests that this maladaptive coping strategy may lead an individual to spend more time online, to be attracted to the experience of being online, and to prefer virtual communication to face-to-face interpersonal communication (49). In our study, the result that adolescents who frequently used avoidant coping in the PIU group preferred more virtual life seems to be consistent with this theory. In addition to all these, our findings confirm previous studies showing that maladaptive cognitions such as avoiding, maladaptive coping, or negative self-image are associated with addictive Internet use (50, 51).

Within the scope of the cognitive-behavioral treatment (CBT) of IA suggested by Young, it was emphasized that individuals’ coping strategies and motivations for internet use should also be considered (52).

The findings presented in this study confirm the necessity of addressing dysfunctional cognitions related to internet use in CBT for IA and applying cognitive restructuring for them. For example, an adolescent with PIU may believe that virtual communication with other people through social networking sites fulfills a social need without experiencing the stress-inducing effects of real interaction. The therapeutic process for this individual should include assessing the maladaptive cognitions that keep him/her online, and cognitive restructuring that allows him/her to see alternative places where he/she can meet his/her social needs in a more functional way. In this way, clinicians can consider coping strategies as a way to prevent or intervene in PIU.

There were several limitations to consider in the current study. We used adolescents’ self-report measures to collect data, which may have led to bias. Future studies may include information from different information sources such as parents, teachers, etc. to strengthen the reliability of the findings. It was not possible to differentiate between specific activities related to the use of social media (e.g. social media used, e.g. instagram, facebook). Future studies should focus on the detailed examination of specific activities and addictive behaviors related to social media. Finally, due to the cross-sectional nature of the study design, causal relationships between variables could not be revealed. Therefore, a longitudinal research paradigm should be applied in future studies.

## Conclusion

Despite these limitations, we concluded that our study is the first to concurrently examine the relationship between problematic internet use and social media addiction, escapism and coping skills. The current study suggests that assessment of social media addiction, escapism, and negative coping styles may be useful mechanisms for clinical formulations of PIU. In other words, our results will provide a better understanding of the PIU clinic, by providing information in terms of factors that may pose a risk for PIU, evaluating the motivations for internet use that may cause PIU, identifying negative coping skills and maladaptive thoughts that may be associated with PIU. These findings provide targeted and actionable practical references to formulate prevention and intervention strategies for PIU in adolescents. Setting time limits on adolescents’ use of social media, determining their motivations for social media use (escape from the real world, preferring virtual life, virtual pleasure, etc.) and developing coping strategies may contribute to the prevention and intervention of PIU.

## Data availability statement

The raw data supporting the conclusions of this article will be made available by the authors, without undue reservation.

## Ethics statement

The studies involving humans were approved by Ataturk University, Faculty of Medicine, Clinical Research Ethics Committee approval number: B.30.2.ATA.0.01.00/418). The studies were conducted in accordance with the local legislation and institutional requirements. Written informed consent for participation in this study was provided by the participants’ legal guardians/next of kin.

## Author contributions

EY: Conceptualization, Data curation, Formal analysis, Investigation, Methodology, Validation, Writing – original draft, Writing – review & editing. MA: Conceptualization, Data curation, Formal analysis, Investigation, Methodology, Writing – original draft, Writing – review & editing. AB: Conceptualization, Data curation, Formal analysis, Investigation, Methodology, Writing – review & editing, Writing – original draft. BB: Conceptualization, Data curation, Investigation, Writing – review & editing, Writing – original draft. BT: Conceptualization, Data curation, Methodology, Supervision, Writing – review & editing, Writing – original draft. SA: Conceptualization, Data curation, Investigation, Methodology, Writing – review & editing, Writing – original draft. İU: Conceptualization, Data curation, Investigation, Methodology, Supervision, Writing – review & editing, Writing – original draft. EA: Conceptualization, Data curation, Investigation, Writing – review & editing, Writing – original draft. GY: Conceptualization, Data curation, Methodology, Supervision, Writing – review & editing, Writing – original draft. AÇ: Conceptualization, Data curation, Formal analysis, Investigation, Methodology, Writing – review & editing, Writing – original draft. HF: Conceptualization, Data curation, Investigation, Methodology, Writing – review & editing, Writing – original draft.
